# Predictors of central and general obesity in Iranian preschool children: which anthropometric indices can be used as screening tools?

**DOI:** 10.1186/s12887-022-03365-4

**Published:** 2022-05-31

**Authors:** Farzaneh Mardali, Mahdyieh Naziri, Mohammad Hassan Sohouli, Somaye Fatahi, Fatemeh Sadat Hosseini-Baharanchi, Mihnea-Alexandru Găman, Farzad Shidfar

**Affiliations:** 1 Department of Nutrition, School of Public Health, Iran University of Medical Sciences, Tehran, Iran; 2 Student of Research Committee, Faculty of Public Health Branch, Iran University of Medical Sciences, Tehran, Iran; 3 Department of Biostatics, Faculty of Health, Iran University of Medical Sciences, Tehran, Iran; 4 Department of Clinical Nutrition and Dietetics, Faculty of Nutrition and Food Technology, National Nutrition and Food Technology, Research Institute Shahid Beheshti University of Medical Sciences, Tehran, Iran; 5 Pediatric Gastroenterology, Hepatology and Nutrition Research Center, Research Institute for Children’s Health, Shahid Beheshti University of Medical Sciences, Tehran, Iran; 6 Minimally Invasive Surgery Research Center & Department of Biostatistics, School of Public Health, Iran University of Medical Sciences, Tehran, Iran; 7 Faculty of Medicine, Department of Hematology, Center of Hematology and Bone Marrow Transplantation, ‘Carol Davila’ University of Medicine and Pharmacy, Bucharest, Romania, Fundeni Clinical Institute, Bucharest, Romania

**Keywords:** Obesity, Overweight, Conicity index, Ponderal index, Body mass index, Waist circumference, Waist to hip ratio

## Abstract

**Aim:**

To compare the ability of anthropometric indices [waist-to-hip ratio (WHR), waist-to-height ratio (WHtR), neck-to-height ratio (NHR), conicity index (CI), body adiposity index (BAI), tri-ponderal mass index (TMI) and body mass index (BMI)] and,measuerments like neck(NC), hip(HC) and waist circumferences to predict overweight and obesity in Iranian preschool children.

**Materials and Methods:**

A total of 498 Iranian preschool children were included in this case–control study conducted in Tehran, Iran. The participants were selected using the stratified random sampling procedure based on gender and school. Using sex-based receiver operating curve (ROC) analysis, we compared the area under the curve and defined the cut-off points for detecting central and general obesity for each index in order to identify the most suitable tools in predicting obesity.

**Results:**

Boys had significantly higher values for NC, WC, WHR, NHR, CI, TMI and BMI as compared to girls, whereas BAI and HC were higher in girls. The area under the curve was calculated for all the possible predictors of central obesity, i.e., NC (0.841–0.860), WC (0.70–0.679), HC (0.785–0.697), WHR (0.446–0.639) and CI (0.773–0.653) in boys and girls, respectively. And according to the ROC curve analysis, BMI (0.959–0.948), TMI (0.988–0.981), WHtR (0.667–0.553) and NHR (0.785–0.769) were predictors of general obesity and NC (0.841–0.860) as predictor of central obesity in boys and girls, respectively. The optimal cut-off points for TMI (13.80–15.83), NC (28.68–27.5) and for other anthropometric indices were estimated in both boys and girls.

**Conclusion:**

TMI and NC seem to predict general and central obesity in Iranian preschool children.

## Introduction

Childhood overweight and obesity, along with global obesity, are some of the most important public health threats worldwide but have received insufficient attention until now [1]. The World Health Organization reports show that overweight and obesity rates in children under the age of 5 and in the 5–18 years age group are 38 and 380 million, respectively [2]. Moreover, this widespread obesity epidemic has augmented the burden of non-communicable diseases [3]. For example, it was estimated that, in 2014, the cost of medicine for a 10-year-old child diagnosed with obesity *versus* a normal-weight child of the same age would cost the United States healthcare system about $12,660—$19,630 [4].

Scientists have proven that overweight and obesity are potential risk factors for the development of cardiovascular disease in later life [5]. As we are faced with an increasing prevalence of chronic disorders, e.g., hypertension, diabetes mellitus, non-alcoholic fatty liver disease and others, there has been a recent focus on detecting which subjects are at risk of developing pediatric overweight and (or) obesity [6, 7].

Routine screening in children should be considered by healthcare providers in a more targeted and serious way. The early assessment of the risk of overweight and obesity in children and adolescents requires practical, widely available, easier to implement and more accurate methods than the existing ones [8–10]. Although magnetic resonance imaging (MRI) [11] and computed tomography (CT) [12] have been considered the gold standard techniques due to their accuracy, they require high costs, are not publicly available to all populations, pose the risk of radiation and their routine use in low/middle income countries is not practical [13]. Over the last decade, researchers have become increasingly interested in anthropometric measurements, e.g., waist or hip circumferences, as potential screening tools for obesity pediatric units [14]. Moreover, neck circumference has been recently assessed in the evaluation of cardiovascular risk and trunk obesity [15, 16]. Although the BMI is the most widely used indicator of weight-for-height status in all age and sex groups [17], unfortunately it is unable to distinguish between lean and fat mass [18]. On the other hand, some studies have shown that, in some children and adults, a normal BMI can co-exist with abdominal obesity [19]. Moreover, waist-to-hip ratio (WHR) predicts the risk of cardio-metabolic diseases, metabolic syndrome and dyslipidemia in children and adults, respectively [20–22]. It seems that the evaluation of waist circumference (WC) along with height and weight can predict the conical shape changes of the body in humans [23]. Although WC is a good indicator of visceral fat, it cannot determine the total body fat [24]. Nowadays, several indices, e.g. the conicity index (CI) [25], the ponderal index [26] and the body adiposity index (BAI) [27], have attracted the attention of researchers worldwide, as they can be easily computed from variables such as height, weight and hip circumference and are feasible indicators of the percentage of body fat and abdominal obesity, respectively [28, 29]. According to the existing evidence, the assessment of visceral fat is more valuable in the evaluation of cardiovascular risk, as it is linked to the distribution of fat mass than the total fat mass [30]. Therefore, indices that can be computed from waist and hip measurements can be more relevant in our clinical practice. In addition, as WC is usually measured relatively to height, misclassification may occur in the anthropometric assessment of tall or short people [30, 31]. However, it reflects abdominal obesity, insulin resistance and cardiovascular risk more accurately [32, 33], and thus the employment of more sensible indices, i.e., the conicity index (CI), tri-ponderal mass index (TMI) and the body adiposity index (BAI) is required. A cross-sectional study conducted in China in 2019 illustrated that the WHR, WHtR and abdominal fat percentage are indicators of abdominal obesity in children aged 6 to 9 years [34]. In a study involving Brazilian children, Nascimento reported that the TMI was a more accurate tool in the prediction of obesity [26]. Given the importance of timely and accurate screening of childhood obesity, it seems that, in different societies, various tools need to be evaluated and compared to identify which are the most reliable instruments in the prediction of this disorder. The aim of our study was to compare the ability of the BMI, TMI, neck-to-height ratio (NHR), BAI, WHtR, NC, WC, hip circumference (HC), CI and WHR ratio in the prediction of central and general obesity in Iranian preschool children.

## Methods

### Participants

This present case–control study was conducted during 2018–2019 in preschool children from Tehran, Iran. A number of 245 normal-weight and 253 children with overweight and obesity from all over Tehran were included in the study. Using the random sampling method, a total of 20 schools of Tehran were randomly selected. The number of schools selected for sampling was equal in both sexes. Matching were done based on class and sex. All the participants were invited to partake in anthropometric measurements. We included preschool children who were singletons and were 6 years of age. The children, who were born prematurely, were twins or resulted from a multiple birth, or who were suffering from growth hormone disorders, metabolic disorders (hypothyroidism, hyperthyroidism, and diabetes), heart disease, renal dysfunctions or bone diseases were excluded from the study. Based on world health organization growth chart,the body mass index (BMI) percentiles for age in both sexes were calculated and the children were divided into two groups: overweight/obese and normal-weight. All children with overweight and obesity were grouped as obese. This study was approved by the research council and ethics committee Iran University of Medical Sciences, Tehran, Iran. Written informed consent was obtained from all patients prior to participation.

### Anthropometry measurement

A trained nutritionist measured the weight and height of children under standard conditions. The children were asked to wear light clothing and remove their shoes in order to have their weights and heights assessed. Standing weight and height were evaluated using a electronicdigital scale (to the nearest 0.5 kg) and a portable stadiometer (to the nearest 0.5 cm) manufactured both by SECA (Hamburg, Germany), respectively. Several circumferences, e.g., waist circumference (WC) at the umbilical level, hip circumference at the widest part of the buttocks, and the neck circumference (NC) in between mid-cervical spine and mid-anterior level, were measured using an Seca brand anthropometric tape (to the nearest 0.1 cm) in a relaxed position. The measurements were repeated 2–3 times and the mean of the results was calculated.

### Calculation of indices and ratios

We calculated the WHR as WC (cm) divided by height (in cm), the WHtR as WC (in cm) divided by hip circumference (cm), and the NHR as NC (in cm) divided by height (in cm). The BMI and TMI were estimated by dividing the weight (in kg) to the squared-height (in m^2^) and the weight (in kg) to cubed-height (in cm^3^), respectively. The CI was calculated based on WC, weight and height using the Valdez's formula [35]. The BAI was computed from the hip circumference and the height based on the formula reported by Aarbaoui et al. [36], taking into account the over/under estimation of body fat in pediatrics. The employed formulas are depicted below:

$$\mathrm{Body}\ \mathrm{Adiposity}\ \mathrm{Index}\ \left(\mathrm{BAI}\right):\kern0.5em \mathrm{hip}\ \mathrm{circumference}\ \left(\mathrm{cm}\right)/{\mathrm{height}}^{0.8}\ \left(\mathrm{m}\right)$$

$$\mathrm{Tri}-\mathrm{ponderal}\ \mathrm{Mass}\ \mathrm{Index}\ \left(\mathrm{TMI}\right):\mathrm{weight}\ \left(\mathrm{kg}\right)/{\mathrm{height}}^3\ \left({\mathrm{cm}}^3\right)$$

$$\mathrm{Conicity}\ \mathrm{Index}\ \left(\mathrm{CI}\right):\mathrm{waist}\ \left(\mathrm{m}\right)/0.109\surd \left[\mathrm{weight}\ \left(\mathrm{kg}\right)/\mathrm{height}\ \left(\mathrm{m}\right)\right]$$

$$\mathrm{Body}\ \mathrm{Mass}\ \mathrm{Index}\ \left(\mathrm{BMI}\right):\mathrm{weight}\ \left(\mathrm{kg}\right)/{\mathrm{height}}^2\ \left({\mathrm{m}}^2\right)$$

### Statistical analysis

The statistical analyses were performed using the statistical software package IBM SPSS Statistics for Windows, version 20.0. The variables were tested for normality using the Kolmogorov–Smirnov test. The descriptive analyses were performed using frequencies (95% confidence intervals, CI) and medians (interquartile ranges) and the comparison of medians between two independent groups by the Mann–Whitney test. The Pearson’s χ^2^ test was used to compare the frequencies of WC, WHtR, NC, HC, WHR, TMI, BAI and CI. The receiver-operating characteristic (ROC) curves were constructed to evaluate the ability of anthropometric measurements as obesity predictors. In addition, the cut-off points for excess central fat were estimated using the best sensitivity and specificity for the different measurements. The hypotheses were tested at a 5% significance level.

## Results

### Characteristics of subjects

In this case–control study, boys represented 48% and 52.8% of the obese and normal-weight preschool children, respectively. Of the 253 children in the case group, the prevalence of overweight and obesity was 62% and 38%, respectively. The mean age of the participants was 6.38 years. The averages of all measured indices in cases *versus* controls were: BMI (20.82 *versus* 15.79 kg/m^2^), NC (29.17 *versus* 26.12 cm), HC (73.79 *versus* 68.54 cm), WC (61.38 *versus* 57.36 cm), CI (1.13 *versus* 1.21), PI (17.45 *versus* 13.38), WHR (0.832 *versus* 0.835) and WHtR (0.51 *versus* 0.48) (data not shown).

The anthropometric indices of the preschool children stratified by sex are represented in Table 1. As compared to boys, girls showed a tendency for lower values of the following indices: weight, height, NC, WC, HC, WHR, WHtR, CI and PI. However, HC and BAI were higher in girls *versus* boys (*P* < 0.05 for all).

**Table 1:** The anthropometric characteristics of the participants stratified based on the presence of obesity/normal-weight

Sex, group	Obese		*P*-value*	Normal-weight		*P*-value*	*P*-value#
	Boys	Girl		Boys	Girls		
**BMI**	20.83 ± 2.65	19.78 ± 2.94	< 0.001	15.79 ± 0.94	14.79 ± 0.8	< 0.001	< 0.001
**NC (cm)**	29.65 ± 2.483	28.72 ± 2.34	< 0.001	26.41 ± 1.98	25.80 ± 1.486	< 0.001	< 0.001
**Weight (kg)**	30.34 ± 4.80	29.50 ± 4.88	< 0.001	22.35 ± 2.40	21.86 ± 2.16	< 0.001	< 0.001
**Height (cm)**	120.5 ± 6.49	119.0 ± 5.48	< 0.001	118.87 ± 5.24	117.56 ± 5.24	< 0.001	< 0.001
**WC (cm)**	62.09 ± 6.52	60.70 ± 6.816	0.01	57.72 ± 3.16	56.32 ± 5.56	0.04	0.02
**HC (cm)**	71.93 ± 6.56	73.65 ± 6.99	< 0.001	67.85 ± 3.47	69.31 ± 3.93	< 0.001	< 0.001
**WHR**	0.839 ± 0.046	0.824 ± 0. 057	0.02	0.813 ± 0.0 68	0.795 ± 0. 094	0.04	0.01
**WHtR**	0.525 ± 0.0503	0.510 ± 0.0541	< 0.001	0.486 ± 0.0349	0.450 ± 0. 0533	< 0.001	< 0.001
**NHR**	24.66 ± 2.44	23.20 ± 2.45	< 0.001	22.26 ± 1.89	20.99 ± 1.62	< 0.001	< 0.001
**BAI**	25.71 ± 5.49	26.10 ± 5.70	< 0.001	21.16 ± 3.50	22.96 ± 3.76	< 0.001	< 0.001
**CI**	1.13 ± 0.099	1.12 ± 0.093	0.01	1.01 ± 0.081	0.99 ± 0.012	0.01	0.03
**TMI**	17.34 ± 5.48	16.57 ± 2.59	< 0.001	13.31 ± 0.99	12.46 ± 0.43	< 0.001	< 0.001

*BMI* body mass index, *NC* neck circumference, *WC* waist circumference, *HC* hip circumference, *WHR* waist-to-hip ratio, *WHtR* waist-to-height ratio, *NHR* neck-to-height ratio, *BAI* body adiposity index, *CI* conicity index, *TMI* tri-ponderal mass index

Continuous data are presented as mean ± SD and compared using the independent sample-test

### Performance of anthropometric measurements

**Fig. 1:**
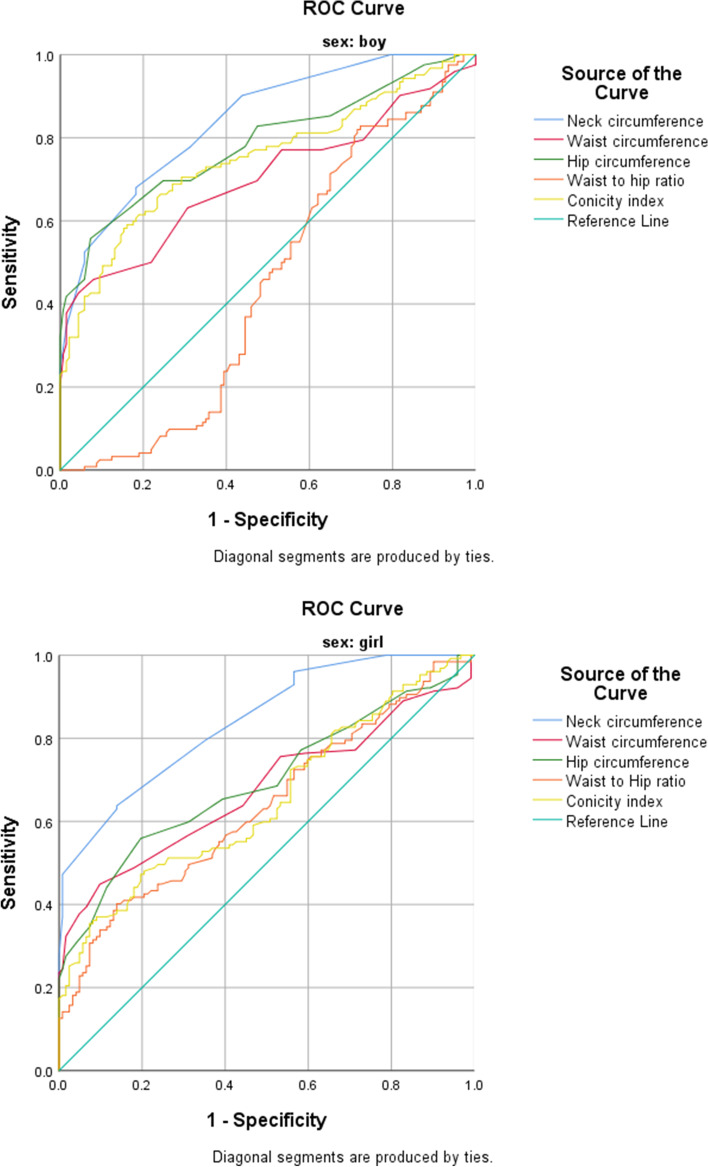
Receiver operation curve for the prediction of central obesity in Iranian preschool children. The relative abilities of neck circumference, waist circumference, hip circumference, waist to hip ratio, and conicity index are compared to normal-weight children in both sexes

**Fig. 2:**
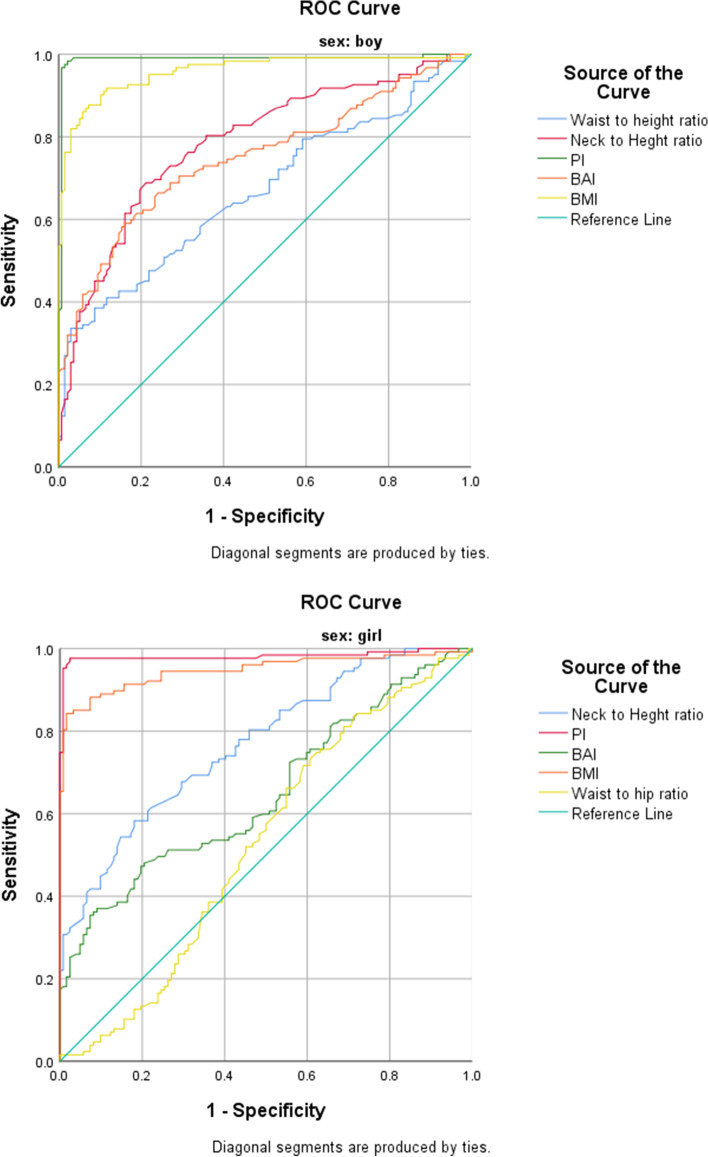
Receiver operation curve for the prediction of general obesity in Iranian preschool children. The relative abilities of tri-ponderal index, body mass index, neck-to-height ratio, body adiposity index and waist-to-height ratio are compared to normal weight children in both sex

The sex-based receiver-operating characteristic curves for all the anthropometric indices employed as predicting tools for the detection of pediatric central and general obesity are represented in Table 2 and Figs. 1–2. In addition, the cut-off points are specified for the all reported variables. ​The ROC curves showed that, in both sexes, the anthropometric indices related to central obesity, i.e., NC, WC, CI and HC were predictors of obesity in 6-year-old children, with the exception of WHR in boys. Moreover, the ROC curves revealed that TMI, BMI, NHR and BAI were predictors of central obesity in both sexes. However, the AUC for WHtR was reported only for boys.

**Table 2:** Sex-based comparison of the Receiver-Operator Characteristic curves for various anthropometric indices evaluated as predictors for central and general obesity among preschool children

		Anthropometric index	AUC	Cut-off value	Sensitivity	Specificity	95% Confidence Interval		*P*-value
							Lower bond	Upper bound	
Boys	Central obesity	NC	0.841	28.68	80.3	63.5	0.794	0.887	< 0.001
		HC	0.785	68.51	77.9	55.5	0.728	0.842	< 0.001
		WC	0.700	58.52	69.7	52.6	.634	.766	< 0.001
		WHR	0.446	-	-	-	0.375	0.517	0.35
		CI	0.773	1.17	75.2	68.7	0.742	0.821	< 0.001
	General obesity	BMI	0.959	17.55	99.2	20.4	0.935	0.983	< 0.001
		TMI	0.988	13.80	96.7	68.6	0.971	0.992	< 0.001
		BAI	0.749	22.14	73.3	35	0.688	0.810	< 0.001
		NHR	0.785	22.74	80.3	35.8	0.729	0.841	< 0.001
		WHtR	0.667	0.83	73.1	56	0.601	0.734	< 0.001
Girls	Central obesity	NC	0.860	27.5	79.5	35.2	0.795	0.884	< 0.001
		HC	0.697	70	68.8	60.7	0.795	0.884	< 0.001
		WC	0.679	57.50	64.1	63.6	.612	.746	< 0.001
		WHR	0.639	0. 80	62.5	51.6	.571	.707	< 0.001
		CI	0.653	1.14	68.9	70.1	0.601	0.712	< 0.001
	General obesity	BMI	0.948	17.10	97.7	99.2	0.862	97.3	< 0.001
		TMI	0.981	15.83	94.5	63.1	0.718	0.977	< 0.001
		BAI	0.651	22.83	64.4	76.7	0.584	0.719	0.01
		NHR	0.769	22.46	73.2	60.7	0.712	0.826	< 0.001
		WHtR	0.530	-	-	-	0.571	0.707	0.12

This table shows the sex-based comparison of the Receiver Operator Characteristic curves for various anthropometric indices evaluated as predictors for central and general obesity among preschool children

Overall, we discovered that, of all the general obesity indices, TMI and BMI were the best predictors of general obesity based on their AUC values, both in boys (0.988 and 0.959, respectively) and in girls (0.981 and 0.948, respectively). In addition, NC was a good predictor of central obesity in both boys and girls (AUC: 0.85 and 0.84, respectively).

## Discussion

In the current population-based study, we demonstrated that the TMI was a better predictor as compared to the BMI in identifying general obesity in pediatric subjects. Moreover, the NC was a suitable tool in screening for central obesity in preschool children. To the best of our knowledge, our study is the first attempt to compare anthropometric indices in the detection of pediatric obesity in Iranian children. In addition, for the first time, the CI, BAI and TMI were computed and evaluated in a population of preschool Iranian children.

Nowadays, due to the increasing prevalence of obesity, researchers have focused their attention also on the period of adipose rebound (ages 5 to 7 years) [37] during which the screening for pediatric obesity using accurate methods might be of paramount importance to prevent its development [38]. Therefore, we suggest that the screening be done more carefully and precisely during this time frame. Thus, it is necessary to identify which screening tools are the most accessible, inexpensive and can be employed by medical practitioners worldwide in different health settings [39].

Our results demonstrated that the BMI and the TMI were the best predictors of pediatric obesity. Similar findings have also been documented by Lorenzo et al. [40], Nascimento et al. [26] and Khoshhali et al. [41]. For example, Lorenzo et al. [40] conducted a cross-sectional study in Italian preschool children and discovered that both the TMI and the BMI were able to anticipate the development of obesity in children aged 12 to 18 years. Similarly, Nascimento et al. [26] reported that the TMI was a predictor of obesity in Brazilian children aged 2 to 5 years. Based on the results of the CASPIAN-V study conducted in Iran, Khoshhal et al. [41] concluded that the BMI and the TMI had medium predictive value for the installment of the metabolic syndrome in subjects aged 12 to 18 years. We observed that, in the prediction of obesity, the optimal TMI and BMI cut-off points were 13.83 and 17.55 in boys, respectively, and 15.83 and 17.10 in girls, respectively.

Similarly to Filgueiras et al. [42], we indicated that the CI is an appropriate indicator of fat distribution in children. It is also noteworthy to mention that differences in the CI may occur due to race or ethnicity or may vary between pediatric age groups and adulthood [43]. Thus, other complementary indices are needed to assess its ability to predict the development of overweight and obesity in children [44]. Our findings delineate that both the NC and the neck-to-height ratio were moderate predictors of pediatric obesity. The same conclusion has also been reached by Nimawat et al. [45], Nafiu et al. [46] and Taheri et al. [15]. For instance, Nimawat et al. [45] evaluated Nigerian children and reported a positive correlation between the NC and the BMI, reinforcing that the measurement of the NC is a simple, accurate and quick method in screening and classifying childhood obesity. The neck-to-height ratio has also been explored to detect obstructive sleep apnea syndrome [47] and has been proven to reflect body fat distribution and to be associated with the presence of metabolic disturbances, hypertension and other cardiovascular disorders [48]. Our results showed that boys have a greater WC *versus* girls. Stupnicki et al. [49], McCarthy et al. [50] and Ashwell et al. [51] have also compared WC in boys and girls. Stupnicki et al. [49] assessed a cohort of Polish children, indicating that boys had smaller WC *versus* girls. Furthermore, a large cohort study from the United States of America conducted by Li et al. [52] illustrated that girls had higher WC *versus* boys irrespective of age. In addition, in the present study, we noted that the WHtR, which has been employed in several studies as a predictor of pediatric and adult obesity, was a predictor of obesity in boys alone. A possible explanation of this phenomenon can be related to the differences in the distribution of body fat between boys and girls [53].

In the current study, we discovered that the AUC for WHR was above the baseline only in girls (girls: 0.639 and boys: 0.446). We suggest that this finding might be explained by the sex-related differences in the skeletal structure of the children enrolled in our research, as well as a greater hip circumference in girls *versus* boys which might indicate a higher percentage of fat in girls. Interestingly, in the recent study on pediatric subjects published by Turcotte et al. [54], several genetic factors, i.e., the HOXC13 rs1443512 and GS genes, influenced the WHR in Mexican girl only. This finding may be due to the effects of gender differences on the incidence genetic polymorphisms that influence WHR. In another research, it was proposed that the WHtR was representative of the total body fat, while WHR was more predictive of the abdominal obesity *versus* other indices, e.g., WHtR, WC or HC [55].

We reported that the BAI was higher in obese *versus* normal-weight children in both sexes. In addition, the ROC curve showed a moderate prognostic value of BAI for the risk of obesity in children (boys: 0.749 and girls: 0.651). This finding was in line with the papers published by Aarbaoui et al. [36], Yu et al. [56] and Filgueiras et al. [42]. For example, Filgueiras et al. [42] reported, based on the results of a cross-sectional study, that the AUCs for BAI were higher-than-expected AUCs both sexes in terms of predicting obesity in Brazilian children and adolescents. As previously suggested, the estimation of the body fat percentage can be conducted using advanced tools such as the dual X-ray absorptiometry (DXA) technique which provides accurate results. However, it has several disadvantages and many of researchers tend to employ more convenient and acceptable methods to estimate fat mass, e.g., the BAI.

Prior studies have described that changes occur in the body composition before puberty, particularly in preschool children from different populations [57]. In the study of Horvat et al. [58], Croatian children aged 6.5 years experienced an increase in fat mass and a muscle mass loss during a 5-year period (1998–2003).

Our study has several strengths. The main strength of this research is that we evaluated and compared various practical anthropometric indices which are easy to calculate, inexpensive and come with negligible errors. Moreover, we assessed, for the first time, the TMI, NC-to-height ratio and BAI of preschool Iranian children. One of the limitations of the present study is the lack of comparison of fat percentage with precision instruments such as DXA, thus the misclassification of some subjects as obese may lead to errors in the interpretation of our findings, and one of the important limitation in this study were lack of assessing anthropometric indices with gold standard methods like magnetic resonance imaging. we suggest that in future studies, gold standard methods such as DEXA and tomography be used to determine body fat percentage. Because of financial constraints and limitations, we were unable to use these methods and as an another limitation of our study,the design of the study was case–control, and it seems that other methods, such as cohort and interventional design, could better predict childhood obesity based on anthropometric methods and obesity related indexes."

In addition, it would have been better to compare a wider age range of the pediatric population.

We suggest that, in future studies, other obesity assessment tools, e.g., WC-to-weight and mid-upper arm circumferences can be used in wider age ranges to improve the available obesity screening tools.

## Conclusion

In Iranian preschool children, the BMI and the TMI are the best predictors of general obesity, whereas the NC is the best predictor of central obesity, as compared to a wide range of anthropometric indices.

## Data Availability

Data is available upon request from the corresponding author for the article due to privacy / ethical restrictions*.*
